# Molecular Evidence for Gender Differences in the Migratory Behaviour of a Small Seabird

**DOI:** 10.1371/journal.pone.0046330

**Published:** 2012-09-27

**Authors:** Renata J. Medeiros, R. Andrew King, William O. C. Symondson, Bernard Cadiou, Bernard Zonfrillo, Mark Bolton, Rab Morton, Stephen Howell, Anthony Clinton, Marcial Felgueiras, Robert J. Thomas

**Affiliations:** 1 Cardiff School of Biosciences, Cardiff University, Cardiff, South Glamorgan, Wales, United Kingdom; 2 Bretagne Vivante – Société pour l'Étude et la Protection de la Nature en Bretagne (SEPNB), Brest, Brittany, France; 3 Institute of Biodiversity, Animal Health, and Comparative Medicine, Glasgow University, Glasgow, Scotland, United Kingdom; 4 RSPB - The Royal Society for the Protection of Birds, Sandy, Bedfordshire, England, United Kingdom; 5 Sanda Island Bird Observatory, Argyll, Scotland, United Kingdom; 6 A Rocha – Associação Cristã de Estudo e Defesa do Ambiente, Mexilhoeira Grande, Algarve, Portugal; Pennsylvania State University, United States of America

## Abstract

Molecular sexing revealed an unexpectedly strong female bias in the sex ratio of pre-breeding European Storm Petrels (*Hydrobates pelagicus*), attracted to playback of conspecific calls during their northwards migration past SW Europe. This bias was consistent across seven years, ranging from 80.8% to 89.7% female (mean annual sex ratio ± SD = 85.5% female ±4.1%). The sex ratio did not differ significantly from unity (i.e., 50% female) among (i) Storm Petrel chicks at a breeding colony in NW France, (ii) adults found dead on beaches in Southern Portugal, (iii) breeding birds attending nest burrows in the UK, captured by hand, and (iv) adults captured near a breeding colony in the UK using copies of the same sound recordings as used in Southern Europe, indicating that females are not inherently more strongly attracted to playback calls than males. A morphological discriminant function analysis failed to provide a good separation of the sexes, showing the importance of molecular sexing for this species. We found no sex difference in the seasonal or nocturnal timing of migration past Southern Europe, but there was a significant tendency for birds to be caught in sex-specific aggregations. The preponderance of females captured in Southern Europe suggests that the sexes may differ in migration route or in their colony-prospecting behaviour during migration, at sites far away from their natal colonies. Such differences in migration behaviour between males and females are poorly understood but have implications for the vulnerability of seabirds to pollution and environmental change at sea during the non-breeding season.

## Introduction

Many species of bird exhibit marked differences between the sexes in aspects of their behaviour, including their foraging behaviour and migration strategies. These differences in foraging behaviour can potentially lead to differences in migration strategies with males and females migrating at different times, travelling by different migration routes, or travelling to and from different wintering grounds [1], [2]. Ultimately this can result in a complete segregation of sexes during migration, having a great impact on population dynamics of many bird species, including through differential mortality [3]. Identifying and investigating sex-differences in migration behaviour is important for our understanding of species' ecology and conservation, but for monomorphic species such studies are hampered by the difficulty of identifying the sex of individuals, particularly outside the breeding season. Previous studies have attempted to address this problem by using morphometric methods such as discriminant function analysis, but such methods can only be reliably applied to species that exhibit a considerable degree of sexual dimorphism. For minimally dimorphic species (such as many storm petrels) only a small proportion of individuals can be sexed with confidence [4]–[6]. As a result, there is a lack of information for such species on sex-differences in behaviour in general, and on migration strategies in particular. Very few studies have addressed sex-specific differences in seabird foraging and ranging behaviour outside the breeding season. To our knowledge, within the Procellariiformes there are only three such studies; two of which were based on stable isotope signatures among various procellariid species [7], [8] and the other used geolocator tracking devices to describe sex differences in migration routes of Balearic Shearwaters (*Puffinus mauretanicus*) [2].

The need to study species throughout their life cycle has been increasingly emphasised as more studies demonstrate the importance of carry-over effects of non-breeding processes into breeding productivity and population dynamics (see e.g., [9]–[11]). Molecular sexing methods now allow accurate sexing of individuals of even complete monomorphic species outside the breeding season (see e.g., [12], [13]), and in this study we applied molecular diagnostics to study differential migration patterns in a highly monomorphic seabird species: the European Storm Petrel (*Hydrobates pelagicus*).

*H. pelagicus* is the smallest Atlantic seabird (∼25 g), and a long-distance migrant between the breeding colonies in the north-east Atlantic and the wintering areas in the south Atlantic and Indian oceans, off southern Africa [14]. Despite their small size, *H. pelagicus* are long lived pelagic seabirds (longevity record = 35 years 9 months [15]) with delayed reproductive maturation. Females lay one large egg per year, which both adults incubate. Both adults also feed the chick for about two months, until shortly before the chick is ready to fledge [16].

Like other Hydrobatidae, *H. pelagicus* normally come inshore only at night [17], and pre-breeding birds can readily be attracted into mist-nets using nocturnal playbacks of sound recordings of conspecific nesting calls (first described in 1980 [18]). These playback calls are effective for catching *H. pelagicus* during their summer northwards migration, even at locations in SW Iberia, far from the nearest known colonies [14], [19]. Most of the birds caught with this method are aged 2–4 years, returning northwards in the years before establishing a breeding site/mate, in order to prospect for these and potentially make their first breeding attempts [20], [21]. Breeding birds are usually not strongly attracted to playbacks of nesting calls since they tend to keep the same mate and nest site between years and therefore cease to prospect for these once they are acquired. *H. pelagicus* are not commonly present at the Atlantic colony sites before the age of two and they usually only start breeding between the ages of three to five years old [21]. Little is known about their movements during the period before they begin returning to the colonies but most do not migrate northwards to Europe during their first year [20]. Breeding birds typically arrive at the colonies in March – May and egg-laying takes place between late-April to mid-August, although there is considerable latitudinal variation in the timing of breeding [22].

Like other storm petrels, *H. pelagicus* show little sexual dimorphism [4] (but see a recent study on sexual dimorphism in the Mediterranean subspecies *H. p. melitensis* [23]). Breeding birds can be sexed at certain times of the year on the basis of cloacal morphology or breeding behaviour [24], [25], but nevertheless little is known about sex-differences in the behaviour and ecology of storm petrels, such as dietary preferences, foraging strategies, migration routes and natal site-fidelity. This lack of knowledge is most marked for the long period when birds are away from the breeding colonies, because of the difficulties involved with observing, catching and sexing the birds during the non-breeding season. Several previous studies have tested for differences between the sexes in the foraging behaviour of other storm petrel species during breeding (see e.g., [7], [8], [26]) but only one study found a difference in the strategies used by male and female of Wilson's Storm Petrel (*Oceanites oceanicus*), which was only apparent in years of food shortage [26].

Molecular sexing techniques now enable migrating playback-lured *H. pelagicus* to be accurately sexed, providing novel insights into the behaviour and ecology of this pelagic seabird away from the breeding colonies. Instead of the X and Y chromosomes found in mammals, birds possess Z and W sex chromosomes, with males being homogametic (ZZ) and females being the heterogametic (ZW) sex. Most species of birds can be sexed with a simple PCR reaction based on size differences of the introns present in both the CHD1-W and CHD1-Z genes (the W- or Z-linked genes coding for the chromodomain-helicase-DNA-binding protein), which are found in most extant non-ratite birds [27]–[29].

The majority of molecular sexing studies have used DNA extracted from blood samples obtained relatively invasively. However, molecular sexing can also be achieved using DNA obtained from a single feathers or faecal sample (see e.g., [30], [31]) - but note a recent study supporting the use of blood versus feather samples [32]. Feathers are becoming more widely used for molecular sexing of birds, but the use of DNA obtained from faecal samples has mainly been explored in mammals.

Using molecular sexing from feathers and faeces, the aims of our study were: (i) To examine gender differences in extent and timing of northward migration into Europe of *H. pelagicus*; (ii) To investigate whether the sex ratios observed among samples of storm petrels caught on migration in Southern Europe are consistent with those obtained at other parts of the annual and life-history cycle; (iii) To examine whether there was evidence of gender grouping in the migratory passage of birds, as a probable indication of sexual segregation at sea [1].

## Methods

### Fieldwork

**Figure 1:**
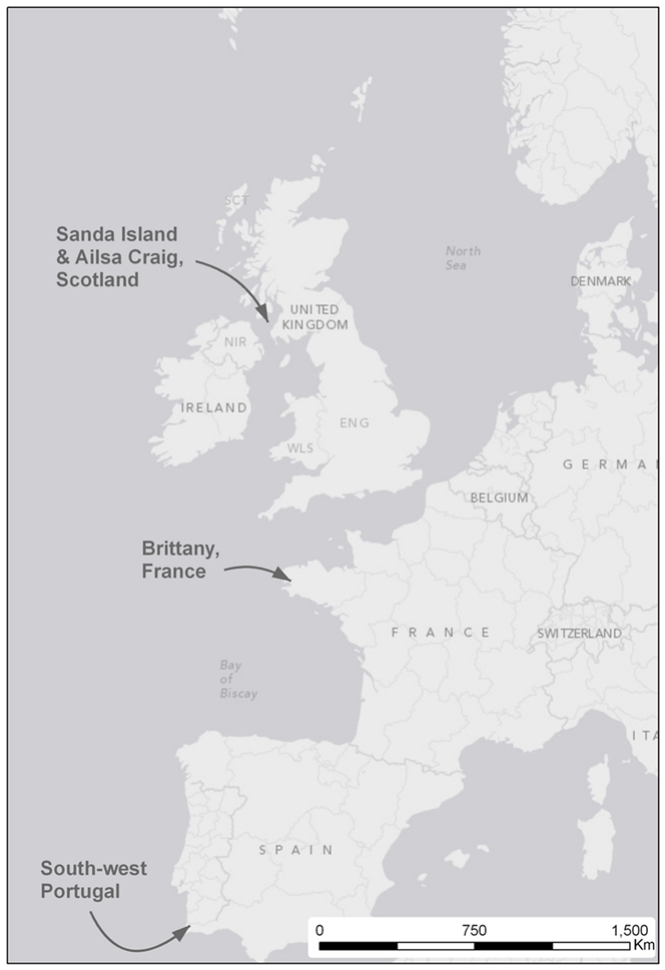
Field sites location. European Storm Petrels were sampled on migration (Portugal), at the breeding colonies (adults - Sanda Island and chicks - Brittany) and near a breeding colony (Ailsa Craig).

*H. pelagicus* were caught in mist-nets at the base of a sea-cliff on the south west coast of Portugal (37° 04′ N, 8° 47′ W), mainland Europe's most southerly location (Fig. 1), using playbacks of the calls that the males perform from their nest sites (usually referred to as the ‘purr’ call [22], [33]). Playback calls took place from dusk (2200 BST) to dawn (0500), within the period mid-May to late June, in all years from 2003–2009. This sampling period spans the main period during which migrating storm petrels can be attracted to playback calls in Southern Europe [19]. European Storm Petrels sampled using playback calls at this field site originate almost entirely from birds originating from the Atlantic, with only a very small number of vagrants (<1% of the total catch) from the non-migratory Mediterranean sub-species (*Hydrobates pelagicus* melitensis) [33].

Two sound recordings of storm petrel “purr calls” were used as playback calls: (i) a recording obtained from the British Trust for Ornithology during the 1990s and (ii) track 11 of disc 1 in the Roche CD collection [34]. The recording-locations of both of these recordings were unknown. These tracks were played on Technika MP series MP3 players coupled to a Martley Megaphone 600 at a sound pressure level of ∼70 dB, and were clearly audible at a distance of approx. 400 m offshore (personal observations). Males respond more strongly than females to playbacks of these burrow calls in terms of calling in reply to the playbacks when inside the nest burrows [35], but previous studies using playback calls of burrow calls to mist-netted storm petrels in or near breeding colonies have found that there is no apparent sex bias in the birds attracted (see Table 1).

**Table 1:** Previously published sex ratio data for *Hydrobates pelagicus*.

Location	Year	Reference	At a colony?	Capture method	Playback used?	Sexing method	Males	Females	Sex ratio: % female	Sig. different from unity?*
Throughout marine range	Prior 1977	[22] (Cramp and Simmons 1977)	At colonies & at sea	Various	Mainly no	Museum skins dissection	20	25	56	No
Skomer, Wales	1981 & 1982	[35] (James 1984)	Yes	Breeders taken on nest	No	Cloacal inspection	43	39	48	No
Skomer, Wales	1982	[36] (James 1983)	Yes	Mist nets	Yes	Discriminant analysis (wing+tail)	31	26	46	No
Skomer, Wales	1982	[36] (James 1983)	Yes	Mist nets	No	Discriminant analysis (wing+tail)	23	20	47	No
Skomer, Wales	1982	[36] (James 1983)	Yes	Breeders taken on nest	No	Cloacal inspection	26	20	43	No
St. Kilda, Scotland	1983	R.W. Furness *In* [37] (Fowler et al. 1986)	Yes “loose colony”	Mist nets	No	Dissection	11	10	48	No
Yell, Shetland	1983 & 1984	[37] (Fowler et al. 1986)	No (but colony on same island)	Mist nets	Yes	Laparoscopy	21	28	57	No

The time of capture was noted (to the nearest minute) and birds were processed in the order in which they were captured. Each bird was ringed and its age determined (as first-year or older than first-year, based on the abrasion and shape of the primary flight feathers [20]). Biometric measures were taken (see details in Supporting Information S1) and between one and four breast feathers (most commonly two) were collected from each bird and kept in a paper envelope at room temperature. All the birds were processed at the site where they were caught, and were released shortly after capture.

We also acquired equivalent samples from *H. pelagicus* breeding locations in the NE Atlantic (Fig. 1): In July 2005, breeding birds attending nest burrows during daytime on Sanda Island, Scotland (55° 16′N, 5° 34′W), were captured by hand. In August 2006, playback was carried out close to the breeding grounds, on Ailsa Craig, Scotland, UK (55° 15′N, 5° 6′W), using the same procedures as those used in Southern Europe, including using exactly the same sound recordings to attract storm petrels into mist nets. At both of these sites, one breast feather was collected from each bird and kept in a paper envelope at room temperature. Faecal samples were collected from chicks at colonies in Brittany, France (48° 23′N, 4° 57′W) during the 2005–06 breeding seasons and stored in 80% ethanol.

In addition, *H. pelagicus* found dead on beaches in southern Portugal (37°07′N 08°36′W) following severe storms in January 1996, were collected for anatomical sexing. On dissection, females were identified by the presence of the single ovary on the left side, and males by the presence of a testicle on each side. Unfortunately these corpses subsequently became decomposed and molecular sexing could not be carried out on them for this study.

### Molecular Sexing

DNA from feathers was isolated using an adaptation of the Chelex extraction method [38]. The barbs towards the base of each feather were removed and approximately 5 mm of the calamus was cut off. 50 µl of distilled H_2_O and 20 µl of InstaGene™ Matrix (BioRad) were added to each sample. The samples were then incubated at 50°C for 30 minutes, followed by 8 minutes at 100°C. DNA from faecal samples was isolated using the QIAGEN® Stool Mini Kit, following the manufacturer's standard protocol. In order to find the best primer combination for this species, preliminary primer testing was performed using primers P8/P2 [27], 1237L/1272H [28], 2550F/2718R [29], P8/M5 [39] and 2550F/TuWR/TuZR [40]. Our comparisons showed that the most effective primer pair for separating male and female *H. pelagicus* was 2550F/2718R [29]. These primers proved to be efficient at a wide range of temperatures and provided the greatest separation of bands (∼200 bp), easily differentiated on a simple agarose gel.

All PCRs included two negative controls prepared with distilled water to test for possible contamination. A gradient PCR was first performed in order to optimise the annealing temperature. One feather extraction and two faecal extractions were used for each temperature gradient PCR. These PCR reactions were performed on a BioRad PTC-225 DNA Engine® Peltier Thermal Cycle PCR machine (45°C to 60°C). The optimum annealing temperatures, obtained from these gradient PCRs, were 50°C for the feather samples and 47.5°C for faecal samples. We sexed 30 individuals (15 males and 15 females) using both feathers and faeces, to compare the results obtained with the two types of samples and check for their consistency. Furthermore, each male result was always repeated at least twice (giving a total of three consistent results) and about 25% of all female results were repeated at least once (giving a total of two consistent results).

Amplifications from feather extractions were made with a standard PCR, carried out in accordance with the study where the primers were originally published [29], using 1 µl of DNA template (∼10 ng/µl). Those from faecal extractions were performed using a Multiplex kit, carried out in 20 µl reactions containing 1× of QIAGEN® Multiplex PCR Master Mix, 0.2 µM of each primer and 3 µl of DNA template (∼3 ng/µl). The thermal conditions were 95°C for 15 min, 35 cycles of 95°C for 1 min, annealing temperature for 1 min 30 s, 72°C for 1 min 30 s, and a final extension at 72°C for 10 min. All reactions were carried out using an Applied Biosystems GeneAmp® PCR System 9700 PCR machine. Samples were run on 2% weight/volume agarose gels stained with ethidium bromide, unless specified otherwise.

### Statistical Analyses

Chi-square tests were used to test for deviation from the expected 1∶1 sex ratio, except for cases in which one or more expected values were less than five, in which case Fisher's exact test was used. Analyses of biometric measures are described in Supporting Information S1. Most of the analyses were carried out using the statistical software packages SPSS (version 16.0 SPSS Inc.) and R (version 2.13.2) [41]; exceptions were the Fisher's exact tests, which were computed at http://www.langsrud.com/fisher.htm, and binomial confidence intervals, which were calculated using a Bayesian calculator available at: www.causascientia.org/math_stat/ProportionCI.html. Significance thresholds were set at *P* = 0.05. Note that the *P*-values presented in our tables are not corrected for multiple comparisons [42], [43]. Means ± 1 SD are presented throughout the text.

General Linear Models (GLMs) were used to test for sex differences in capture date (controlling statistically for differences in year and time of night), and to test for sex differences in the time of night of capture (controlling statistically for year and capture date). A runs test was carried out using R, to test the hypothesis that storm petrels captured using playback calls in Southern Europe were captured in sex-specific groups. Given that unequal numbers of males and females were captured, we used the simulation-based method for a “biased coin” runs test [44] to test whether the observed number of runs of consecutive same-sex individuals (within each night) was significantly different from the number of such runs expected if individuals of the two sexes occurred in a random sequence.

## Results

### Sex differences in biometrics and consistency of molecular sexing from feathers and faecal samples

Although some differences in biometric measures were found between males and females *H. pelagicus* (Table S1), a morphological discriminant function analysis failed to provide satisfactory discrimination between the sexes (see details in Supporting Information S1).

For the molecular sexing, the overall proportion of feather samples that yielded DNA of sufficient quality to give a sexing result was 94%, while the equivalent proportion from faecal samples was 71%. When sexed from faecal samples, birds previously identified as female from the feathers often amplify only one of the two fragments, Z or W. When the W-fragment (female specific) is evident, birds can still be sexed with confidence. However, when only the Z-fragment (shared by males and females) is visible, females will be misidentified as males. Accordingly, 100% of birds sexed as male from feathers were also sexed as male from faeces, but 43% of females sexed from feathers were initially sexed as male from faeces. This proportion dropped to 14% after repeating the sexing procedure for each apparent male result three times (see Methods). To take into account this error in sexing from faecal samples, we should assume that 14% of male results (between one and two individual birds) from the chicks sexed from faeces (see above) could be females. This would still result in a non-significant sex ratio bias (χ^2^ = 2.793, df = 1, *P* = 0.095). For those birds sexed from feathers only, less than 3% of individuals initially classified as males were reclassified as females after the three repeats and none of the initial female results were reclassified as males in subsequent testing.

### Sex ratios of adult *H. pelagicus*

A strongly female biased sex ratio (85.0±4.1% female) was found in the sample of birds captured in Southern Europe in all seven years (Table 2) with no significant differences in sex ratio among years (χ^2^ = 11.794, df = 6, *P* = 0.07) and no significant trend in sex ratio over the seven years (Spearman's rank correlation: *r_s_* = +0.214, *n* = 7 years, *P* = 0.645). The vast majority of the birds caught were at least two years old, with only 0.01% of individuals being of either undetermined age, or definitely in their first year. Among the birds that were sexed, many carried rings from other countries, or were later recaptured in other countries; a female-biased sex-ratio was also found in these birds regardless of the country where they were previously ringed or subsequently recaptured (Table 3).

**Table 2:** Sex ratios of *Hydrobates pelagicus* adults and chicks in different locations and years.

Year	Female	Male	Total	Sex ratio (% female)	95% CI limits (% female)	Chi-squared test for deviation from unity (1∶1)
Playback-lured birds, Portugal						
2003	83	12	95	87.4	79.2–92.6	?^2^ ** = **53.1, *P*<0.001
2004	81	17	98	82.7	73.9–88.9	?^2^ ** = **41.8, *P*<0.001
2005	122	16	138	88.4	82.0–92.7	?^2^ ** = **81.4, *P*<0.001
2006	105	25	130	80.8	73.1–86.6	?^2^ = 49.2, *P*<0.001
2007	93	11	104	89.4	82.0–94.0	?^2^ = 65.6, *P*<0.001
2008	90	22	112	80.4	72.0–86.6	?^2^ = 41.3, *P*<0.001
2009	236	27	263	89.7	85.5–92.8	?^2^ = 166.1, *P*<0.001
All years combined	810	130	940	86.2	83.8–88.2	?^2^ = 491.9, *P*<0.001
**Storm-killed birds, Portugal** (1996)	6	12	18	33.3%	16.3–56.6%	?^2^ = 6.096, *P* = 0.297
**Playback-lured birds, Scotland** (2006)	14	16	30	46.7%	30.2–64.0%	?^2^ = 0.133, *P* = 0.715
**Hand-caught birds, Scotland** (2005)	15	17	32	46.9%	30.8–63.6%	?^2^ = 0.125, *P* = 0.724
**Chicks, France** (2005+2006)	17	12	29	58.6%	40.6–74.5%	?^2^ = 0.862, *P* = 0.353

All samples were sexed using DNA extracted from feathers, except for the storm-killed birds in Portugal (sexed by dissection) and the chicks sampled in France (sexed using DNA extracted from faeces - see Methods).

**Table 3:** Sex ratio of *Hydrobates pelagicus* controlled in different countries or re-trapped in Portugal.

Location	Males	Females	Sex ratio (% female)	Fisher's Exact test for deviation from unity
Iceland, Norway & Denmark	1	16	94.1	*P* = 0.007
UK & Ireland	13	56	81.2	*P*<0.001
France, Spain & Italy	3	15	83.3	*P* = 0.07
Same-year re-traps in Portugal	0	5	100	*P* = 0.17

A total of 18 dead *H. pelagicus* were recovered from beaches in Portugal in January 1996. These birds were all aged as juveniles (fledged within the preceding five months). Anatomical sexing revealed this sample to be comprised of 12 males and six females, but this apparent male-bias was not significantly different from 50% female (Table 2).

Adult *H. pelagicus* attracted to playback calls in the UK, close to their breeding colonies, using the same sound recordings as used in Southern Europe, also showed a sex ratio that was not significantly different from 50% female (Table 2), suggesting that the sex bias in Southern Europe was not simply an artefact of the use of playback calls. Although this sex ratio was estimated from a relatively small sample of 30 birds, we found that 100 random sub-samples of 30 birds from the much larger Southern Europe sample gave a mean sex ratio (± SE) of 84.7% (±0.80), with only 4% of these sub-samples giving a female bias smaller than 64%, which was the upper 95% confidence interval of the sample captured in the UK. Thus, the apparent difference in sex ratio between birds captured in Southern Europe and the UK does not appear to be an artefact of smaller size of the UK sample.

Breeding birds caught at their nest sites on Sanda Island in Scotland, UK during the incubation period also showed a sex ratio that was not significantly different from 50% female (Table 2). This was expected given that both sexes incubate eggs equally [22]. In the absence of birds of known sex to validate the molecular sexing, this provides useful confirmation of the reliability of the molecular method.

### Sex ratio among *H. pelagicus* chicks

From the chicks examined at the breeding colony in France, nine faecal samples were collected in 2005 and 29 in 2006. In 2005, four chicks were found to be female and three were male (two samples could not be sexed); in 2006, 12 chicks were found to be female and 10 were male (seven could not be sexed). Data from both years were pooled to allow for statistical analysis. This indicated that the observed primary sex ratio of sexable chicks at this breeding colony did not deviate significantly from 50% female (Table 2).

### Evidence of gender grouping in the migratory passage of birds

**Figure 2:**
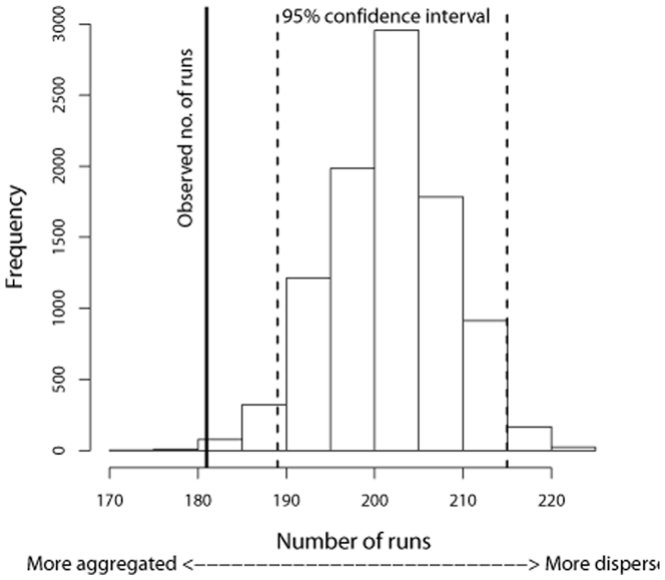
Frequency distribution of the expected number of runs of consecutive same-sex captures. Results based on 10,000 random samples drawn from a population of 116 males and 755 females. The dotted vertical lines show the 95% CI limits of the number of runs expected from a random sequence of males and females (189–215 runs), while the solid vertical line shows the observed number of runs (181). The smaller number of same-sex runs observed than expected indicates a greater degree of aggregation than would be expected if males and females are caught in a random sequence.

Over the 1.5 months of the annual study period, there was no significant seasonal difference in when males and females were captured (mean difference ± SE = males 0.40±0.65 days before females, GLM: *F* = 0.375, df = 1, 926, *P* = 0.540). Similarly, there was no significant difference in the time of night at which males and females were captured (mean difference ± SE = males 0.28±0.17 hours before females, GLM: *F* = 0.280, df = 1, 926, *P* = 0.103). However, a runs test with unequal sample sizes showed that there were significantly fewer “runs” of consecutive catches of birds of the same sex (181 runs), than expected from random sequences drawn from the sample of 116 males and 755 females (*P*<0.01, 99% CI limits = 184–219 runs, Figure 2). A lower number of runs than expected indicates sex-specific aggregation (the extreme case would be all individuals of one sex caught first, followed by all captures of the other sex, giving just two runs of same-sex captures). A higher number of runs than expected would indicate less aggregation than expected from males and females being captured in random sequences (the extreme case would be each successive capture of the 118 males being interspersed with a capture of one or more females, giving a total of (116×2)+1 = 233 runs). The observed number of runs (181) was significantly lower than expected; hence there was a tendency for storm petrels to occur in sex-specific groups as they were trapped in Southern Europe.

## Discussion

The use of feathers as a DNA source to sex European Storm Petrels was highly successful. Considering that a maximum of only three breast feathers were plucked, our opinion is that the impact on the birds was considerably less than would have been caused by blood sampling which requires longer handling times. The storage and preservation of feather samples is also very simple compared to blood samples. Therefore, despite the concerns recently raised on the use of feathers for molecular analysis [33], we believe that breast-feather samples can be a very useful source of DNA for sexing purposes. Overall, the major criticisms made of molecular techniques for sexing birds are related to (i) preferential amplification of the Z-chromosome fragment [45], (ii) the fact that the male is defined by the absence of amplification of the W-chromosome fragment, in other words, by a negative result [46], and (iii) polymorphism in the Z chromosome [45], [47]. Errors related to criticisms (i) and (ii) would result in females being wrongly classified as males, which seems unlikely to have occurred in our study, given the direction of the sex-ratio bias in our main results. Furthermore, primers 2550F/2718R have advantages that minimise such potential sexing errors [45], [47]. The presence of polymorphism in the Z chromosome could lead to the misidentification of males as females. Although primers 2550F/2718R might be more prone to this error compared to other primers, they have the advantage that such polymorphism is also more likely to be detected [45]. In our case, polymorphism was detected with an extra band appearing in about 20% of the samples. This did not interfere with the sex identification. In order to overcome these potential errors, Shizuka and Lyon [48] developed a new W-specific primer (GWR2) to be used in combination with 1237L/1272H. This approach is very promising but we could not test it in this study because it was published after our research had been completed.

The molecular sexing analysis revealed a very strongly female-biased sex ratio among *H. pelagicus* sampled during their northwards migration past the Southern Europe's coast, several hundred kilometres from the nearest known breeding colonies. This sex ratio bias was broadly consistent over the seven years of our study (varying between 81% and 90%), indicating that it is a stable feature of the birds available for capture using playback calls at this location (comprised almost entirely of wandering pre-breeders from the Atlantic).

The highly female-biased sex ratio that we observed among birds attracted to playback calls in Southern Europe is strikingly and consistently different from the approximately 50% sex ratio found among storm petrels of a variety of age classes sampled using a variety of techniques and sexing methods, at or near the NE Atlantic breeding colonies (Table 1). We also found no evidence for any difference in the sex ratios of *H. pelagicus* from different geographical origins recaptured in our study site (Table 3).

Besides any possible differences in migration behaviour between male and female *H. pelagicus*, there could be other explanations for the female sex bias observed in Southern Europe, such as a real sex-ratio bias in the population or an inherently stronger attraction of females to the playback calls. For a sex ratio bias in a population to persist, a consistent bias in the primary sex ratio (amongst eggs/chicks) and/or a sex-specific mortality rate after fledging must be present. The primary sex ratio may be biased in some taxa, including some bird species [49]–[51]. However, such examples are exceptional and most bird populations, especially in monogamous species, exhibit approximately 50% primary sex ratios (reviewed by [52]). Indeed, we did not find any bias in the primary sex ratio among the chicks hatched by *H. pelagicus* breeding at a colony in NW France, suggesting that this is not the explanation for any sex-ratio bias in the adult population.

A female-biased adult sex-ratio could arise from an unbiased primary sex ratio if males suffer greater mortality than females. In contrast to mammals, greater male mortality is very uncommon among birds (reviewed by [51]). However, in one species of petrel (a diving petrel, *Pelecanoides urinatrix*) a significant male biased mortality has been found among storm-killed individuals [53]. The sex-ratio in the sample of *H. pelagicus* killed during winter storms off the Portuguese coast did not differ significantly from unity (though we note that more males than females were killed; see Table 2). Since these birds were found freshly dead in January, they do not belong to the female-biased sample of pre-breeders travelling northwards in May-June, but instead they are likely to be comprised of birds recently fledged from the more northerly breeding colonies (from which birds fledge later than from more southerly colonies). Even if male European Storm Petrels are more likely to be killed by storms, this seems unlikely to give rise to such a highly consistent female-biased sex ratio in all years of our study, since severe-weather mortality is unlikely to occur to such a similar extent in all years. A total of 45 museum skins of European Storm Petrels from throughout the species' range and annual cycle also show an unbiased sex ratio (Table 1) and no significant sex ratio biases were found in any of the previous studies summarized in Table 1. Furthermore, in the present study, the sex ratios among live birds attracted to playback calls near a breeding colony in Scotland and among live birds captured without playback calls at nest sites in Scotland were also unbiased. There is therefore little support for the hypothesis that there is an underlying bias in the sex ratio of the population as a whole.

Another hypothesis accounting for the female-biased sex ratio observed in this study is the possibility that female storm petrels are inherently more attracted to playback of conspecific calls than are males. However, one study found that male European Storm Petrels in nesting burrows responded more strongly than females to playbacks of the “purr” calls [35]. The context of the playback at the nest used in that study, in which males might need to defend their nest from other males, is very different from the context of the playback calls in the present study, and this potential male-bias is not consistent with our finding that use of the same playback calls near a breeding colony resulted in an unbiased sex ratio. Similarly, the two studies presented in Table 1 on sex ratios of *H. pelagicus* caught either at- or close to- a breeding colony with playback calls show no sex ratio bias.

Differences in the migration behaviour of pre-breeders male and female European Storm Petrels are therefore most likely to explain the female bias found among the birds caught on the Southern Europe coast. This also supported by our observation that storm petrels tend to occur in sex-specific aggregations, suggesting some degree of segregation between the sexes at sea. Studies of stable isotope levels in different petrel species outside the breeding season, had revealed evidence for sex- related differences in foraging only in large, sexually size-dimorphic albatrosses and giant petrels [7], [8], suggesting that the type of prey consumed by smaller petrels did not vary greatly between the sexes. However, this does not exclude the possibility of sex-segregation at sea by smaller petrels. Indeed, a recent study found differences in the migratory patterns between males and females of a highly monophorpic, medium size, petrel species, the Balearic Shearwater [2].

Sex differences in the migration behaviour of *H. pelagicus* could take various forms; none of the following hypotheses is necessarily mutually exclusive. A biased sex ratio among pre-breeding petrels captured in Southern Europe could arise if males and females are differentially attracted to the purr call at different times in the year or at different locations. The former could arise if males need to find their burrows earlier in the breeding season than females [54], to which they subsequently attract a female, while the latter could occur if females are more likely to disperse between breeding colonies, and so more willing to investigate breeding locations in Southern Europe, well outside their main breeding range. To our knowledge, no research has investigated these possibilities, but among our sample of wandering pre-breeders, we did not find evidence for gender differences in the timing of passage (at neither the overnight scale nor the seasonal scale).

European Storm Petrels can be active both by day and by night and therefore sex-differences in the diurnal/nocturnal pattern of migration could also make females more likely to come within auditory range of the nocturnal playback calls. We found no difference between males and females in the time of night at which they were captured.

Sex differences in migration strategy could lead to more females than males being present in Southern Europe coastal waters during the May–June study period. There are several potential underlying mechanisms. Females may, for example, start to wonder north at a younger age. Pre-breeding European Storm Petrels attracted to playback calls in the UK have an unbiased sex ratio (Tables 1 and 2), but the observed female bias in this study could arise if these younger females reach as far north as SW Europe but do not wander all the way north to the breeding colonies. The only method available to determine the age of European Storm Petrels in the hand can only distinguish birds in their first year from those older than that [20]. Having more birds ringed as chicks could greatly improve our understanding of age- and sex-specific behaviour.

The sex-bias in the Southern Europe sample could be related to seasonal differences linked to sex-differences in the time of arrival at the breeding colonies. In many migrant species, the breeding males are the first to arrive back on the breeding grounds (protandry), to set up territories or secure a mate (see e.g., [55], [56]). We found no difference between males and females in capture date, indicating that if males really are migrating at a different season than females, then this male migration must take place outside our study period of late May–June. However, from past experience over two decades of catching storm petrels in Southern Europe (including attempts to capture birds throughout the year), it appears that there is a short and variable period of time each year in early-mid Summer when most birds are caught and hardly any birds are caught before or after this period, suggesting that if males really do travel earlier (or later) than females, then they are not responding to the playback calls at these times.

Finally, the sexes could have different migration routes, impossibly related to foraging strategies. For example, one study in Black-legged Kittiwakes (*Rissa tridactyla*) found that 58% of the males made a long-distance pre-breeding movement to an area unexploited by the females [57]. Overall, capturing storm petrels from boats further offshore, and at additional locations, further north and south along the migration route, as well as in the wintering grounds, could greatly improve our understanding of the mechanisms underlying the behavioural differences between males and females.

Although the underlying mechanisms behind this sex bias remain unclear, our findings show the importance of considering sex specific behaviour in interpreting ecological data. For example, sex-differences in migration behaviour may be important in considering the conservation of seabird species away from the breeding colonies, including the habitats that they use across their annual cycles.

## Supporting Information

Supporting Information S1 Sex differences in biometrics of European Storm Petrels (*Hydrobates pelagicus*) attracted to playback calls in Southern Europe.
(DOC)

Table S1 Mean body measurements (mm) and body mass (g) for *Hydrobates pelagicus* caught in Portugal between 1989–2008 (± SE).
(DOC)
