# Supplementary material for: Molecular Evidence for Gender Differences in the Migratory Behaviour of a Small Seabird
Source: PLoS One. 2012 Sep 27;7(9):e46330. doi: 10.1371/journal.pone.0046330 (PMC3459920; doi:10.1371/journal.pone.0046330)
Supplement: Supporting Information S1 — Sex differences in biometrics of European Storm Petrels (Hydrobates pelagicus) attracted to playback calls in Southern Europe. (DOC) [file pone.0046330.s001.doc]

**Molecular evidence for gender differences in the migratory behaviour of a small seabird**

Renata J. Medeiros^1^, R. Andrew King^1*^, William O. C. Symondson^1^, Bernard Cadiou^2^, Bernard Zonfrillo^3^, Mark Bolton^4^, Rab Morton^5^, Stephen Howell^1^, Anthony Clinton^1^, Marcial Felgueiras^6^ and Robert J. Thomas^1^

^1^*Cardiff School of Biosciences, Cardiff University, Cardiff, South Glamorgan, Wales, UK*

^2^*Bretagne Vivante – Société pour l'Étude et la Protection de la Nature en Bretagne (SEPNB), Brest, Brittany, France*

^3^ *Institute of Biodiversity, Animal Health, and Comparative Medicine, Glasgow University, Glasgow, Scotland, UK*

^4^*RSPB - The Royal Society for the Protection of Birds*, *Sandy, Bedfordshire, England, UK*

^5^*Sanda Island Bird Observatory, Argyll, Scotland, UK*

^6^*A Rocha – Associação Cristã de Estudo e Defesa do Ambiente, Mexilhoeira Grande, Algarve, Portugal*

** Current address:* *College of Life and Environmental Sciences, University of Exeter, Exeter, Devon, England, UK*

PLoS One

**Corresponding author:** Renata Medeiros, medeirosmirrarj@cardiff.ac.uk

**Supporting Information**

iiiiiiiiiiiiiiiS1. Sex differences in biometrics of European Storm Petrels (*Hydrobates pelagicus*) attracted to playback calls in Southern Europe

Biometric measures were taken of body mass, wing length, tarsus length (from the depression in the angle of the intertarsal joint to the base of the last complete scale before the toes diverge), culmen length (from the tip of the bill to the feathering at the base of the bill), “bill depth 1” (from the bottom of the mandible to the top of the nostril tube, taken at the depression mid-way along the tube), “bill depth 2” (from the bottom of the mandible to the top of the maxilla taken just anterior to the nostril), head-plus-bill length (from the tip of the bill to the back of the skull) and rump width (the anterior-posterior width of the visible white feathering of rump-patch). Wing length and body mass were the only measures taken during all seven years; the other measures were recorded only from 2006-2009 with the exception of head-plus-bill (recorded from 2006-2008) and rump width (only recorded in 2009).

T-tests were used to compare birds’ measurements between sexes and a discriminant function analysis was used to examine whether birds could be reliably sexed on the basis of morphometric measurements.

On average, males had significantly lower body mass, shorter wings and deeper bills (in terms of bill depth 2) than females, but we found no significant differences between the sexes in measurements of tarsus, culmen, bill depth 1, head-and-bill length, or rump measurements (Table S1). The best discriminant function obtained for the storm petrels based on structural biometrics and using a randomly selected subset of females to equal the sample size of the males, included two variables: the ratio of bill depth 2 to culmen, and the ratio of wing length to tarsus. The resulting function is as follows:

Discriminant score = -0.654*billdepth2/culmen + 0.810*wing/tarsus (Wilks’ Lambda = 0.900, Chi-square = 9.396, *P* = 0.009).

This discriminant function correctly classified only 63% out of the 92 birds that were sexed using molecular techniques; 62% of 45 males and 64% of 47 females. This is not a very useful level of discrimination since in our study population we could obtain a higher proportion of birds correctly sexed (~85%) by simply assuming they were all female.
