# Supplementary material for: Molecular Evidence for Gender Differences in the Migratory Behaviour of a Small Seabird
Source: PLoS One. 2012 Sep 27;7(9):e46330. doi: 10.1371/journal.pone.0046330 (PMC3459920; doi:10.1371/journal.pone.0046330)
Supplement: Table S1 — Mean body measurements (mm) and body mass (g) for Hydrobates pelagicus caught in Portugal between 1989–2008 (± SE). (DOC) [file pone.0046330.s002.doc]

**Molecular evidence for gender differences in the migratory behaviour of a small seabird**

Renata J. Medeiros^1^, R. Andrew King^1*^, William O. C. Symondson^1^, Bernard Cadiou^2^, Bernard Zonfrillo^3^, Mark Bolton^4^, Rab Morton^5^, Stephen Howell^1^, Anthony Clinton^1^, Marcial Felgueiras^6^ and Robert J. Thomas^1^

^1^*Cardiff School of Biosciences, Cardiff University, Cardiff, South Glamorgan, Wales, UK*

^2^*Bretagne Vivante – Société pour l'Étude et la Protection de la Nature en Bretagne (SEPNB), Brest, Brittany, France*

^3^ *Institute of Biodiversity, Animal Health, and Comparative Medicine, Glasgow University, Glasgow, Scotland, UK*

^4^*RSPB - The Royal Society for the Protection of Birds*, *Sandy, Bedfordshire, England, UK*

^5^*Sanda Island Bird Observatory, Argyll, Scotland, UK*

^6^*A Rocha – Associação Cristã de Estudo e Defesa do Ambiente, Mexilhoeira Grande, Algarve, Portugal*

** Current address:* *College of Life and Environmental Sciences, University of Exeter, Exeter, Devon, England, UK*

PLoS One

**Corresponding author:** Renata Medeiros, medeirosmirrarj@cardiff.ac.uk

**Supporting Information**

Table S1. Mean body measurements (mm) and body mass (g) for *Hydrobates pelagicus* caught in Portugal between 1989-2008 (± SE).

|  | **Tarsus** | **Bill depth 1** | **Bill depth 2** | **Culmen** | **Head and Bill** | **Wing** | **Rump** | **Body Mass** |
| --- | --- | --- | --- | --- | --- | --- | --- | --- |
| **Male** | 22.6 ± 0.78  (*n* = 81) | 4.6 ± 0.35  (*n* = 52) | 3.8 ± 0.31  (*n* = 52) | 11.7 ± 0.53  (*n* = 71) | 31.9 ± 0.77  (*n* = 53) | 122.8 ± 2.80  (*n* = 130) | 14.8 ± 2.23  (*n* = 27) | 26.0 ± 2.05  (*n* = 129) |
| **Female** | 22.5 ± 0.71  (*n* = 473) | 4.5 ± 0.26  (*n* = 343) | 3.7 ± 0.22  (*n* = 343) | 11.8 ± 0.76  (*n* = 432) | 31.8 ± 0.65  (*n* = 239) | 123.8 ± 2.55  (*n* = 806) | 14.9 ± 2.18  (*n* = 234) | 26.4 ± 2.30  (*n* = 805) |
| ***t*-test** | *t* =1.57,  df = 552  *P* = 0.118 | *t* = 0.51  df =393  *P* = 0.132 | t = 2.10  df =58.5  ***P* = 0.040** | *t* =1.21  df = 501  *P* = 0.225 | *t* = 0.79  df = 290  *P* = 0.428 | *t* = 4.00  df = 934  ***P* < 0.001** | *t* = 0.26  df =259  *P* = 0.795 | *t* = 2.04  df = 932  ***P* = 0.042** |
